# Vestibular recruitment: new application for an old concept

**DOI:** 10.1016/j.bjorl.2021.04.006

**Published:** 2021-05-07

**Authors:** Roseli Saraiva Moreira Bittar, Raquel Mezzalira, Alice Carolina Mataruco Ramos, Gabriel Henrique Risso, Danilo Martin Real, Signe Schuster Grasel

**Affiliations:** a Universidade de São Paulo, Faculdade de Medicina, Departamento de Otorrinolaringologia, São Paulo, SP, Brazil; b Universidade Estadual de Campinas (UNICAMP), Departamento de Otorrinolaringologia, Campinas, SP, Brazil

**Keywords:** Vestibular recruitment, Vestibular compensation, Caloric test

## Abstract

**Introduction:**

Vestibular recruitment is a sign of hyperexcitability of central vestibular neurons and may be characteristic of peripheral vestibular damage.

**Objective:**

To define the post-caloric recruitment index and its ability to predict the stage of vestibular compensation and peripheral lesion.

**Methods:**

First of all, we demonstrated that larger values in the cold post-caloric stimulation compared to warm stimulation were equivalent to vestibular recruitment observed during the sinusoidal harmonic acceleration test. In the next step, patients with vestibular complaints and asymptomatic controls were submitted to the caloric test. We calculated post-caloric recruitment index for the control group. Among the study group, we analyzed the relation between post-caloric recruitment and unilateral weakness as well as the types of vestibular diagnoses.

**Results:**

Mean post-caloric recruitment was 17.06% and 33.37% among the control and study group, respectively. The ratio between post-caloric recruitment and unilateral weakness was 1.3 in the study group. Among recruiting subjects, no significant difference of unilateral weakness from the lesioned or healthy side was observed. We found no differences in vestibular diagnoses between recruiting and non-recruiting subjects.

**Conclusion:**

Post-caloric recruitment index identified asymmetric vestibular tonus and central compensation. The normal value was established at 17.06%.

## Highlights

• Post caloric recruitment index is the ratio of the angular velocity of the slow phase obtained by cold and warm caloric stimulation of the same ear.
• The normal value was established in 17.06%.
• Post caloric recruitment index is useful to identify the affected ear separately.
• Recruitment suggests that central compensation is not complete yet in individuals with vestibular symptoms and peripheral lesion.

## Introduction

The vestibulo-ocular reflex (VOR) is considered a key tool to detect diseases of the vestibular system. At least three neurons are involved in the basic VOR, including the eighth cranial nerve and the first neuron of the vestibular pathway.^1^ During rest, the neural input of both ears is constant and symmetric. It sustains a stable and symmetric tonus of both vestibular nuclei. A peripheral lesion disrupts the balance between the nuclei. At this point, compensatory mechanisms involving neural plasticity are initiated with the goal of reestablishing the symmetric tonus of the vestibular nuclei known as central compensation.^2^, ^3^, ^4^

When a lesioned nerve that is irresponsive to low-intensity stimuli is able to generate a response within or even above normal limits as the stimulus increases, we call this “neural recruitment”.^5^ Van Egmond coined the term “vestibular recruitment” in 1949.^6^ The author observed that some subjects with vestibular damage did not show responses to low-frequency angular acceleration during the rotatory test, but had normal responses to high-frequency stimulation. Later, other authors demonstrated that in a normal ear the vestibular response is exactly proportional to the angular acceleration stimulus, but shows an exponential increase in damaged ears during the rotary test.^7^, ^8^ Vestibular recruitment was accepted as an objective event that reflects the damaged peripheral information, the balance between the vestibular nuclei and the stage of central compensation.^6^, ^9^ Some authors have used the caloric stimulus to detect vestibular recruitment in a protocol not used in clinical practice.^1^, ^10^

The caloric test is widely used to investigate each labyrinth’s activity separately, and theoretically, is able to detect the damaged side. The classic unilateral caloric stimulation produces asymmetric tonus of the vestibular nuclei and induces a response that is similar to a peripheral afferent lesion with unilateral hypofunction.

Unilateral weakness (UW) is a measure that evaluates whether responses are symmetric between both labyrinths and demonstrates a functional relationship of both ears. It detects asymmetric vestibular information and unilateral hypofunction. The idea is questionable, since in bilateral lesions, unilateral weakness may not exist.

Alternated excitatory (warm) and inhibitory (cold) stimulations of both ears allow us to evaluate the stage of central compensation in patients with vestibular diseases. When VIII nerve afferent information is partially impaired, the warm (excitatory) stimulus will not produce an effective response to trigger the motor neurons.^11^ The deafferented nucleus produces a weak response because the peripheral stimulus is weaker, and the commissural pathways send inhibitory signals from the contralateral healthy nucleus. On the other hand, the cold inhibitory stimulus further reduces the peripheral information and the basal activity of the lesioned nucleus. Therefore, the final response reflects the basal tonus of the healthy side maximized by absent inhibition of the lesioned side. The contralateral healthy nucleus is not inhibited by the commissural pathway and shows a strong response to stimulation.^12^ This asymmetry can only be explained by an increased tonus of the contralateral, healthy vestibular nucleus. In this case we can say that neural recruitment is positive.^1^, ^6^

Physiologic fundamentals show us that an increased ratio between cold and warm in the same ear suggests asymmetric tonus between vestibular nuclei. This asymmetry may result from a lesion of the afferent pathway and gives information about the patient’s stage of compensation. Routine vestibular tests do not include the post-caloric recruitment phenomenon (PCR). In our clinical practice, we observe that patients with PCR show recruitment in the rotatory test, thus indicating impaired vestibular input. This clinical observation demanded further investigation. Therefore, the aim was to observe recruitment of the vestibular system through routine caloric test responses to 30- and 44-degrees’ water irrigation.

## Objectives

Establish normal values for the ratio of the angular velocity of the slow phase (AVSP) obtained by cold and warm stimulation of the same ear named post-caloric recruitment index (PCRI).

Verify if PCRI can predict the stage of vestibular compensation and peripheral damage.

## Methods

This cross-sectional cohort study was approved by the Institution’s Ethic Committee (nº 2.841.376, August 24, 2018). The study was conducted at the outpatient clinic of Neurotology, from 2018 to 2020.

### First phase: testing the hypothesis

We evaluated 21-patients with dizziness of vestibular etiology (vertigo, disequilibrium, unsteadiness) to test the hypothesis that PCR represents vestibular nerve recruitment. The mean angular velocity of the slow phase (AVSP) to cold stimulation was at least 3-degrees larger as compared to the mean AVSP of warm irrigation in all subjects (mean AVSP cold – mean AVSP warm >3). All subjects were submitted to the sinusoidal harmonic acceleration test (SHAT) in a rotatory chair (Nydiag 200, Interacoustics) with test frequencies of 0.04 Hz and 0.16 Hz. The subject sits in a computer-controlled chair that rotates alternately clockwise and counterclockwise in different frequencies.^13^ The test parameters are described in Table 1. Forty-six healthy asymptomatic volunteers were the control group and had the same tests. The results of the initial investigation were used to design the methods of this study.

**Table 1:** Sinusoidal harmonic acceleration test parameters.

Velocity (degrees/second)	Frequency (Hz)	Cycles	Test time (minutes)	Direction
50	0.040	2	1:15	left
50	0.160	5	0:37	left

### Second phase: PCRI calculus

#### Sample

We selected a control group of 133 healthy volunteers without vestibular complaints (77-females and 56-males) to obtain the PCRI value. All subjects performed the caloric test. We only included subjects with no history of dizziness.

The study group was composed of 381subjects with vestibular complaints (262 females and 119 males), recruited from the outpatient neurotology clinic. Clinical complaints included any kind of dizziness with variable duration and evolution. All patients were submitted to the standard clinical investigation protocol: medical history, ENT examination, cranial nerve evaluation, static and dynamic equilibrium tests (Romberg and Fukuda), coordination tests and caloric test.

The inclusion criteria were the same for both groups: age 18 or older; cognitive ability to understand and execute the tests; and sign the informed consent form. Exclusion criteria were cognitive disability; ocular, cervical, rheumatic, and orthopedic diseases that prevented test execution.

#### Methods

We used the Interacoustics VN415 (Denmark) and ICS Chartr 200 Otometrics (Denmark) equipment for the caloric test. Caloric stimuli were delivered by water irrigation at 44 and 30 degrees Celsius in this order: warm left ear, warm right ear, cold left ear, cold right ear. Jongkees formula was used to calculate Unilateral weakness (UW).^14^ UW above 20% was considered abnormal.^15^

The post-caloric recruitment index (PCRI) was defined for the control group (healthy individuals) based on the Jongkees formula, comparing the AVSP bi thermal test results of the same ear: PCRI = (AVSP cold – AVSP warm/AVSP cold + AVSP warm) × 100.

The formula was used for all subjects’ caloric test results. We considered a positive test result when the cold stimulations produced larger values than the warm stimulations. All positive test results were analyzed. These results were used to determine PCRI of each ear in both groups. The mean value obtained among healthy individuals was considered normal PCRI. We also evaluated the UW in both groups. The study group subjects with PCRI above the mean value of the control group sample were considered “recruiting”.

Finally, we analyzed the underlying diagnosis of the study group subjects and the proportion of PCRI positive and negative individuals.

### Statistical analysis

In the first study phase, normal distribution of SHAT was tested using the Kolgomorov–Smirnov test. Mean and standard deviation were submitted to *t*-test.

In the second phase, PCRI value distribution was tested using the Kolgomorov–Smirnov test. Mean and standard deviation of both groups were submitted to *t*-test. Median values and standard deviation were shown in box plots. The Fisher’s exact test was used to study the association between PCRI, caloric test, UW and diagnosis.

Significance was set at alpha = 0.05, Confidence Interval of 95%.

## Results

### First study phase: test of hypothesis

**Figure 1:**
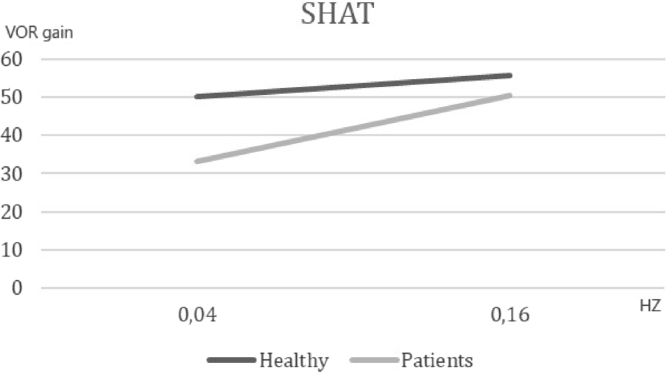
VOR gain at frequencies of 0.04 and 0.16 Hz in healthy individuals and dizzy patients submitted to sinusoidal harmonic acceleration test (SHAT).

The first phase compared 21 symptomatic subjects [(mean AVSP cold) – (mean AVSP warm) >3] and 46 volunteers. The SHAT results are depicted in Fig. 1. We observed a significant difference of mean AVSP gain of the rotatory reflex at acceleration of 0.04 Hz (healthy subjects: 50.06, patients: 33.28, *p* < 0.002). No difference between groups was detected at the frequency of 0.16 Hz (healthy: 55.68, vestibular patients: 50.57, *p* = 0.41). These results support the hypothesis that vestibular recruitment exists among symptomatic subjects.

### Second study phase: PCRI calculus

The second study phase calculated PCRI of 133 individuals (266 ears) without complaints (healthy control group), 77 (56%) females and 56 (42%) males with a mean age of 42 years. Among 266 ears, we selected 147 (55%) with [(mean AVSP cold) – (mean AVSP warm) > 3] to establish mean and standard deviation. Mean PCRI was 17.06%, standard deviation: 12.48, median: 13.51.

**Figure 2:**
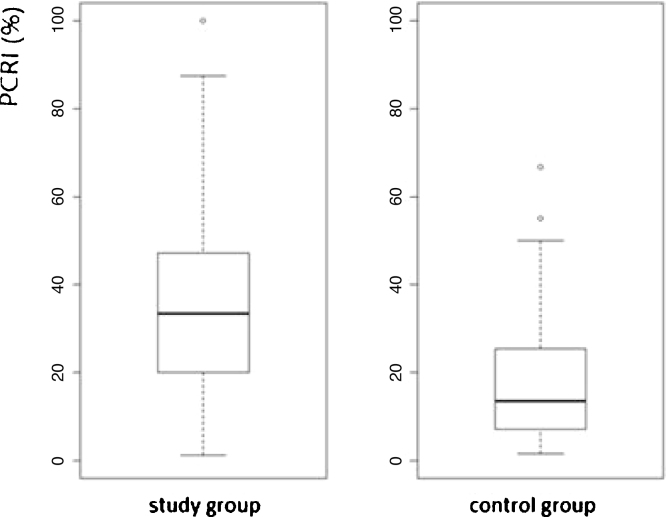
Box plot showing PCRI (%) distribution of 133 healthy individuals (median: 13.51) and 381 patients (median: 28.88), (*p* < 2.2e-16, 95% CI).

The study group consisted of 262 (69%) females and 119 (31%) males with a mean age of 52-years. Among 762-ears, 467 (61%) with mean [(mean AVSP cold) – (mean AVSP warm) > 3] were evaluated. Mean PCRI was 33.37%, SD = 25.56 and median 28.88, and was significantly higher when compared to control group subjects (*p* < 2.2e-16, 95% CI). The values of both groups are shown in a box plot (Fig. 2).

The bi caudal Fisher’s test with 2 × 2 contingency tables was used to analyze the association of UW and PCRI ≥17% in both groups. We found more cases of UW among study group subjects than among healthy controls (*p* = 0.0003). The ratio between PCRI (180) and UW (136) was 1.3(*p* = 0.0001) in the study group. Comparing unilateral to bilateral PCRI, UW was more frequent in unilateral cases (*p* = 0.0023). Analyzing unilateral recruitment and UW, there was no difference between ipsilateral or contralateral UW (*p* = 0.344451). In about 50% of the cases, UW came from the healthy side.

Diagnoses did not differ between study group individuals with recruitment and without recruitment, suggesting no relationship between the specific diagnosis and recruitment (Table 2). There was no significant difference of central recruitment among both groups (*p* = 0.1115).

**Table 2:** Types of diagnoses among study group subjects. REC means higher responses to cold than to warm stimulation; nREC means similar responses to warm and cold stimulation (*p < 0.05).

Diagnosis	REC	nREC	*p*
Migraine	38	26	0.079
Central vestibular syndrome	58	95	0.647
Metabolic/hormonal cochleovestibular disorder	31	25	0.316
Cerebellopontine angle tumor	2	6	0.292
Anxiety/Stress	6	3	0.319
Peripheral (neuritis, BPPV)	8	11	0.814
Hydrops	2	1	0.605
Other cochleovestibular disorders (motion sickness, neurovascular contact)	23	28	0.881
No diagnosis	12	6	0.146
Total of subjects	180	201	

## Discussion

When submitted to rotatory test, subjects with vestibular impairment showed normal responses to high frequency rotation, but significantly lower responses to low frequency rotation when compared to the control group, confirming the recruitment (REC) observed during the caloric test. This result supports that the conventional caloric test identifies “recruiting” patients. So, REC permits the observation of the individual diseased ear with a low-cost and accessible method.

During the caloric test of the damaged labyrinth, the warm stimulus evokes an excitatory current with a decreased response. However, the cold stimulus evokes disinhibits the contralateral nucleus, already with increased tonus, resulting in warm/cold response dissociation. Therefore, post-caloric recruitment is a combination of peripheral and central effects. Type II nuclear cells are highly relevant in this process as they are the only ones with inhibitory potential in the vestibular pathway. These cells are present in the commissural system responsible for the plasticity that rebalances the neuronal activity between both vestibular nuclei. Recruitment suggests that the stimulus intensity and the response of the ocular motor neurons are ruled by central modulation. Normally, a sub-threshold stimulation of the inner ear will not generate a response, but repeated stimuli will sum up the excitatory signals in the vestibular pathways and facilitate central responses. Under pathologic conditions, only stronger stimuli will evoke a response, suggesting that the excitatory threshold of the central vestibular system is elevated.^11^ Following this concept, it is reasonable to suppose that REC indicates some kind of activity of the vestibular system that can disappear after treatment or effective vestibular compensation.

After vestibular compensation, information comes from the healthy vestibular nerve, replaces the lesioned afferences (partially or completely), and regulates both vestibular nuclei.^3^ Rebalancing of the commissural pathways involves both the vestibular nuclei and the cerebellar flocculus. These are adaptive mechanisms of inhibitory (GABA) and excitatory neurotransmitters on the deafferented medial vestibular nucleus cells. Furthermore, adaptation of the inhibitory drive of the flocculus and synaptic reorganization occurs. Static symptoms like spontaneous nystagmus may disappear during the compensation progress. Still, dynamic VOR damage after peripheral lesion may never fully recover, even in the long term.^4^ Hence a peripheral vestibular lesion is able to induce central hyperexcitability.

Caution is necessary when we try to identify in central vestibular disorders the origin of recruitment: central or peripheral. According to Ghosh and Kacker,^10^ when the slow phase angular velocity values are initially elevated and increase rapidly as stimuli increase, system hyperactivity is present and central recruitment is suggested. In this case the lesion affects the inhibitory mechanisms of the vestibular pathways. Post-caloric asymmetry may be the result of hyperactivity when angular velocity is increased or is gradually increasing during the test. A lesion of the vestibular nucleus or intercommissural fibers may mimic recruitment originated from a peripheral lesion.^10^ Morphological analysis of post-caloric nystagmus, absence of the inhibitory effect of ocular fixation or compromised oculomotor activity allow the differential diagnosis.^16^ Peripheral recruitment is only detected when there is hypoactivity after warm stimulation and a disproportionate response after cold stimulation without any central signs.

When recruitment is present in individuals with vestibular symptoms and peripheral lesion, it suggests that central compensation is not complete yet. In fact, we have observed that in our clinical practice recruiting patients usually show good outcomes after adequate treatment of etiological factors and vestibular rehabilitation. Functional VOR recovery can occur after connection of synapses with the partially lesioned hair cells followed by partial or total recovery of synaptic function and, therefore, modulating central compensation.^17^ The CNS responds to input of novel peripheral information that may not be equivalent to the afferent signals prior to the lesion. The reafferentation may prolong the compensation process, thus extending the recruitment period.

Not only vestibular symptoms, but also metabolic and hormonal factors should be addressed to achieve adequate control of disease activity. Moreover, proper indication of exercises is crucial for central compensation.^18^ Recruitment represents a period of “neural activity” trying to reestablish functional balance between both vestibular nuclei. The female hormone cycle, stress, anxiety, and depression may alter the hypothalamic–pituitary-adrenal axis and affect central compensation. Glucocorticoids can modulate synaptic and central function, interact with ionic channels of the cellular membrane, and regulate the activity of neurotransmitters.^4^ Some steroids have negative effects on central compensation, which is why anxious patients may experience prolonged clinical evolution. Likewise, prolonged clinical recovery may be found in patients with premenstrual syndrome, as progesterone and metabolites can modulate the expression of vestibular nuclei GABA receptors.^18^ In these cases, treatment of the hormonal disorder improves patients’ central compensation, reducing the adaptive period of recruitment.

The study group subjects are part of a cross-sectional cohort and had different kinds of diagnosis and duration of vestibular diseases, hence the elevated standard deviation was expected. We considered “recruiting” patients with PCRI values >17%. As shown in Fig. 2, the box plot of mean PCR indicates high variability of means in both groups. The variability found among study group subjects seems to reflect the different stages of the lesion and the progress of central compensation. The elevated standard deviation, even among asymptomatic subjects without history of dizziness, is an interesting finding. This may suggest that the vestibular system is able to maintain postural stability and dynamic performance even with input of asymmetric information. It may occur after infection, inflammation, hormone or metabolic fluctuation, common events during one’s lifetime. The greater the asymmetry between the vestibular nuclei, the higher the PCRI. Among the symptomatic study group subjects, the index refers to the progress of central compensation and does not just describe the “status” of the lesion. It helps to develop treatment strategies for the patient and to predict the prognosis of the vestibular disease.^9^, ^19^

Unilateral weakness (UW) is a parameter commonly used to identify the lesioned labyrinth in a peripheral disease. Unilateral weakness is important, as it indicates the lesioned side before intervention or surgery. Nevertheless, in case of unilateral hyperfunction, the normal side appears to be functionally “weaker”. Among patients with unilateral disease, PCRI was more frequent than UW (1.3–1.0). This finding is expected as the UW formula is based on the evaluation of both labyrinths. Bilateral disease may be overlooked, when both sides show reduced AVSP with non-significant differences to obtain the index.^1^ It is particularly troubling when there is no difference between ipsilateral and contralateral PCRI and UW, so it is not possible to identify the lesioned labyrinth simply through UW, notably in case of surgical indication. PCRI is able to detect dysfunction of each individual ear and avoid inadequate interventions.

For diagnosis we divided the study group in “recruiting” and “non-recruiting” subjects and did not find differences between the groups. Central recruitment was equally observed in both groups. This finding suggests that REC is not related to the syndromic diagnosis, but rather reflects the functional stage of vestibular pathways.

Finally, PCRI may provide valuable information to manage vestibular diseases with no additional cost, time, or inconvenience for the patient in routine diagnostic evaluation. It is useful to identify the affected ear separately and to provide information regarding the progress of central compensation. PCRI is not only useful for diagnosis, but also for clinical management and prognosis of the lesion.

Our results have practical implications for clinical care and further studies are required to confirm the relationship between the vestibular recruitment in the caloric test, peripheral damage, and the stage of vestibular compensation. In the absence of central disease, PCRI calculated from the caloric test, seems to be a low-cost, useful, and practical tool to identify the peripheral lesion.

## Conclusion

The normal value of the PCRI was established at 17.06%. It detected asymmetric vestibular tonus due to peripheral damage and can predict the stage of central compensation.

## Conflicts of interest

The authors declare no conflicts of interest.
